# Food consumption and combined dietary patterns in relation to Crohn’s disease: a case–control study in Saudi Arabia

**DOI:** 10.3389/fnut.2026.1989162

**Published:** 2026-10-23

**Authors:** Manal Malibary, Haytham A. Sheerah, Ahmed Arafa, Mudi H. Alharbi, Norah Eid Aljohani, Maryam M. Alghamdi, Shoroog M. Allogmanny, Amnah N. Yamani, Mustafa M. Shoqeair, Osama Z. Alzeer, Haneen N. Molla, Anas Almofarreh

**Affiliations:** 1 Department of Food and Nutrition, Faculty of Human Sciences and Design, King Abdulaziz University, Jeddah, Saudi Arabia; 2 Food, Nutrition and Lifestyle Research Unit, King Fahd Medical Research Center, King Abdulaziz University, Jeddah, Saudi Arabia; 3 Chief Executive Office, Public Health Authority, Riyadh, Saudi Arabia; 4 Population Health Deputyship, Ministry of Health, Riyadh, Saudi Arabia; 5 Department of Public Health and Community Medicine, Faculty of Medicine, Beni-Suef University, Beni-Suef, Egypt; 6 Clinical Nutrition Department, College of Applied Medical Sciences, Taibah University, Madinah, Saudi Arabia; 7 Center of Innovation in Personalized Medicine, King Fahd Medical Research Center, King Abdulaziz University, Jeddah, Saudi Arabia; 8 Clinical Nutrition Department, King Khalid University Hospital, King Saud University Medical City, Riyadh, Saudi Arabia; 9 The Investment Deputyship, Ministry of Health, Riyadh, Saudi Arabia

**Keywords:** Crohn’s disease, diet, dietary patterns, fast food, inflammatory bowel disease, soft drinks

## Abstract

**Background:**

Dietary factors have been associated with Crohn’s disease (CD), but evidence regarding the role of combined dietary patterns remains limited. We investigated the associations of individual food consumption and broader dietary patterns with CD.

**Methods:**

This case–control study included 219 patients with CD and 395 controls. Intake frequency of selected foods was scored on a four-level scale. Based on the combined consumption of foods classified as risk-promoting or protective, participants were grouped into four dietary patterns: high risk-promoting/low protective, high risk-promoting/high protective, low risk-promoting/low protective, and low risk-promoting/high protective. Associations with CD were assessed using multivariable logistic regression and expressed as odds ratios (ORs) with 95% confidence intervals (CIs).

**Results:**

Patients with CD reported more frequent intakes of fast food, carbonated soft drinks, spicy food, and fruit: ORs (95% CIs) = 4.12 (3.02, 5.63), 3.89 (2.74, 5.52), 1.67 (1.33, 2.09), and 3.48 (2.41, 5.02), respectively. Participants with low consumption of risk-promoting foods had substantially lower CD odds, regardless of whether their consumption of protective foods was low (OR = 0.16, 95% CI: 0.07, 0.35) or high (OR = 0.14, 95% CI: 0.07, 0.27). High consumption of protective foods was not associated with lower CD odds when consumption of risk-promoting foods was also high (OR = 1.15, 95% CI: 0.69, 1.92).

**Conclusion:**

Frequent intakes of fast food, carbonated soft drinks, and spicy food were associated with greater CD odds. Dietary patterns characterized by low consumption of risk-promoting foods were associated with substantially lower CD odds.

## Introduction

Crohn’s disease (CD) is a chronic inflammatory bowel disease (IBD) that can affect any part of the gastrointestinal tract and is characterized by periods of active disease and remission. Common manifestations of CD include abdominal pain, diarrhea, rectal bleeding, weight loss, and fatigue. CD frequently develops in young adults and may require prolonged medical treatment, repeated hospitalization, or surgery (1). Its long-term course can also impair physical and psychological well-being and quality of life and increase healthcare costs (1–4).

The epidemiology of CD has changed considerably over recent decades. While the disease has historically been concentrated in North America and Europe, its burden is increasing in regions where CD was previously less common (5, 6). This pattern is also evident in Arab populations. A systematic review from the Arab region estimated an annual CD incidence of approximately 1.46 per 100,000 persons and reported increasing rates of IBD over time (7). Rapid urbanization and socioeconomic development across the region have been accompanied by major changes in lifestyle, including a shift away from traditional diets toward more Westernized eating habits (8, 9).

The development of CD reflects interactions between genetic, immunological, microbial, and environmental factors (10, 11). Diet is of particular interest because, unlike genetic susceptibility, it can potentially be modified. Dietary exposures may affect intestinal inflammation through changes in the gut microbiota and the intestinal environment (12–14). Prospective evidence has linked inflammatory dietary patterns and greater consumption of Western and ultra-processed foods and carbonated beverages with a higher risk of CD (15–19), while higher fiber intake and greater adherence to Mediterranean or other healthy dietary patterns have been associated with lower risk (20–23). Increased consumption of processed foods, fast food, and sugar-sweetened beverages has become increasingly common in Arab countries as part of the ongoing nutrition transition (8, 9), making dietary factors particularly relevant to the study of CD in this population.

Using hospital-based data collected in Saudi Arabia, we previously examined several individual foods and beverages in relation to CD (24–28). These studies focused on specific dietary exposures. However, foods are consumed as part of broader dietary patterns rather than in isolation; therefore, evaluating them jointly may provide information beyond that obtained from individual-food analyses. In addition, previous analyses relied on grouped consumption categories, limiting assessment across the full range of intake frequency. Sex-specific associations were also not examined consistently, despite reported differences in environmental exposures and IBD susceptibility between men and women (29, 30).

The present study therefore extended these previous analyses by using the full ordinal consumption scale, evaluating combined dietary patterns, and conducting sex-stratified analyses. We assessed associations between individual food consumption scores and CD and examined dietary patterns based on joint levels of selected food groups.

## Materials and methods

### Study design, setting, and population

We conducted a hospital-based case–control study using clinical and questionnaire data from a private polyclinic in Riyadh, Saudi Arabia, between January 2009 and December 2017. Cases were adults aged ≥18 years with a new diagnosis of CD. Controls were adults attending the same clinic during the same period for gastrointestinal conditions other than IBD, primarily gastroenteritis (*n* = 344; 87.1% of controls), with smaller numbers diagnosed with gastroesophageal reflux disease, irritable bowel syndrome, hemorrhoids, or gallstone disease. Individuals with previous or current evidence of IBD, gastrointestinal malignancy, polyposis, or diverticulosis were not eligible as controls. Participants with incomplete dietary data were excluded as well.

### Confirmation of Crohn’s disease

Patients with symptoms of IBD were initially assessed clinically and underwent urine and stool testing, complete blood count, and measurement of inflammatory markers. Those with findings suggestive of IBD proceeded to colonoscopy. Biopsy specimens collected during colonoscopy were evaluated histopathologically, and CD cases were included only when the diagnosis was supported by both colonoscopic and histopathological findings.

### Dietary and covariate assessment

Dietary information was obtained using a self-administered questionnaire completed before the results of laboratory investigations, colonoscopy, and histopathological examination were available. Participants were asked to report their usual frequency of consuming fast food, carbonated soft drinks, spicy food, fruits, vegetables, dairy products, coffee, and tea during the period preceding the onset of their current gastrointestinal symptoms. Periods of fasting, including Ramadan, were excluded. For each food or beverage item, the response categories were monthly or less, weekly, twice weekly, and daily. The questionnaire assessed consumption frequency but did not collect information on portion size, total energy intake, number of meals, or nutrient intake. Therefore, the dietary variables represent frequency of consumption rather than quantitative intake. Information on age, sex, and smoking behavior was obtained from the questionnaire. Weight and height were measured at the clinic for body mass index (BMI) calculation. Laboratory measurements of inflammatory markers, including white blood cell (WBC), erythrocyte sedimentation rate (ESR), and C-reactive protein (CRP), were obtained from medical records.

### Statistical analysis

Continuous variables were summarized using means and standard deviations (SDs), while categorical variables were described using numbers and percentages. Differences between patients with CD and controls were tested using the independent-samples *t*-test for continuous variables and the chi-square test for categorical variables.

Dietary frequencies were coded from 1 to 4, with 1 representing monthly or less, 2 weekly, 3 twice weekly, and 4 daily consumptions. Each food score was analyzed as an ordinal variable, so the estimated OR represented the change in the odds of CD for a one-category increase in consumption frequency. Binary logistic regression was used to calculate odds ratios (ORs) and 95% confidence intervals (CIs). Model I included age and sex, Model II additionally included BMI and smoking, and Model III further included WBC, ESR, and CRP. As a sensitivity analysis, each dietary item was also dichotomized as daily versus non-daily consumption (twice weekly, weekly, or monthly or less). This dichotomization was used because some individual frequency categories contained few participants, resulting in sparse data and unstable estimates when all four categories were modeled separately. Effect modification by sex was formally assessed by including multiplicative interaction terms between sex and each dietary exposure in the regression models, and analyses were repeated separately for men and women, with adjustment for age, BMI, smoking, and inflammatory markers.

A risk-promoting dietary score was calculated as the mean of the four ordinal consumption scores for fast food, carbonated soft drinks, spicy food, and fruits. Similarly, a protective dietary score was calculated as the mean of the four ordinal consumption scores for vegetables, dairy products, coffee, and tea. Each food item was scored from 1 to 4, corresponding to monthly or less, weekly, twice weekly, and daily consumption, respectively. Therefore, both the risk-promoting and protective dietary scores ranged from 1 to 4. For each score, an average value of ≥3 was classified as high consumption, whereas a value <3 was classified as low consumption. The two classifications were then combined to generate four dietary patterns: high risk-promoting/low protective, high risk-promoting/high protective, low risk-promoting/low protective, and low risk-promoting/high protective.

All analyses were conducted using IBM SPSS Statistics for Windows, version 22.0 (IBM Corp., Armonk, NY, USA), and statistical significance was defined as a two-sided *p*-value < 0.05.

## Results

### Participant characteristics

The study included 219 patients with CD and 395 controls. Patients with CD were younger than controls (33.8 ± 7.8 vs. 37.6 ± 8.9 years, *p*-value < 0.001) and had a lower mean BMI (22.6 ± 4.7 vs. 27.2 ± 6.4 kg/m^2^, *p*-value < 0.001). Men represented 69.4% of the CD group and 64.8% of the control group, with no significant difference between the groups (*p*-value = 0.248). Smoking patterns were also similar (*p*-value = 0.219). Elevated counts of ESR (29.3% vs. 20.6%, *p*-value = 0.015) and CRP (20.9% vs. 13.6%, *p*-value = 0.018) were more common among patients with CD. Elevated WBC count was also more frequent in the CD group, although the difference was not statistically significant (4.2% vs. 1.7%, *p*-value = 0.082) (Table 1).

**TABLE 1:** Sociodemographic and clinical characteristics of patients with Crohn’s disease and controls.

Characteristics		Crohn’s disease *n* = 219	Control *n* = 395	*P*-value
Age (years), mean ± SD		33.8 ± 7.8	37.6 ± 8.9	<0.001
Sex, %	Men	69.4	64.8	0.248
	Women	30.6	35.2	
Body mass index (kg/m^2^), mean ± SD		22.6 ± 4.7	27.2 ± 6.4	<0.001
Smoking, %	No	79.9	79.3	0.219
	<10 cig/day	3.2	1.0	
	10–20 cig/day	11.4	12.9	
	>20 cig/day	5.5	6.8	
Elevated white blood cell, %		4.2	1.7	0.082
Elevated erythrocyte sedimentation rate, %		29.3	20.6	0.015
Elevated C-reactive protein, %		20.9	13.6	0.018

### Dietary consumption

Patients with CD reported more frequent daily consumption of fast food (61.6% vs. 19.7%), carbonated soft drinks (65.8% vs. 24.8%), and spicy food (41.1% vs. 30.9%), as well as more frequent twice-weekly consumption of fruits (78.5% vs. 57.0%) (Figure 1). They also showed higher mean consumption scores for fast food (3.53 ± 0.67 vs. 2.61 ± 0.92, *p*-value < 0.001), carbonated soft drinks (3.61 ± 0.59 vs. 3.01 ± 0.74, *p*-value < 0.001), spicy food (3.17 ± 0.81 vs. 2.85 ± 0.87, *p*-value < 0.001), and fruits (3.08 ± 0.49 vs. 2.83 ± 0.66, *p*-value < 0.001). Mean consumption scores were lower among patients with CD for vegetables (3.49 ± 0.59 vs. 3.65 ± 0.50, *p*-value = 0.001), dairy products (3.67 ± 0.57 vs. 3.76 ± 0.43, *p*-value = 0.026), and coffee (3.21 ± 0.85 vs. 3.35 ± 0.80, *p*-value = 0.038), while tea consumption did not differ significantly (3.14 ± 0.83 vs. 3.27 ± 0.80, *p*-value = 0.052) (Figure 2 and Table 2).

**FIGURE 1:**
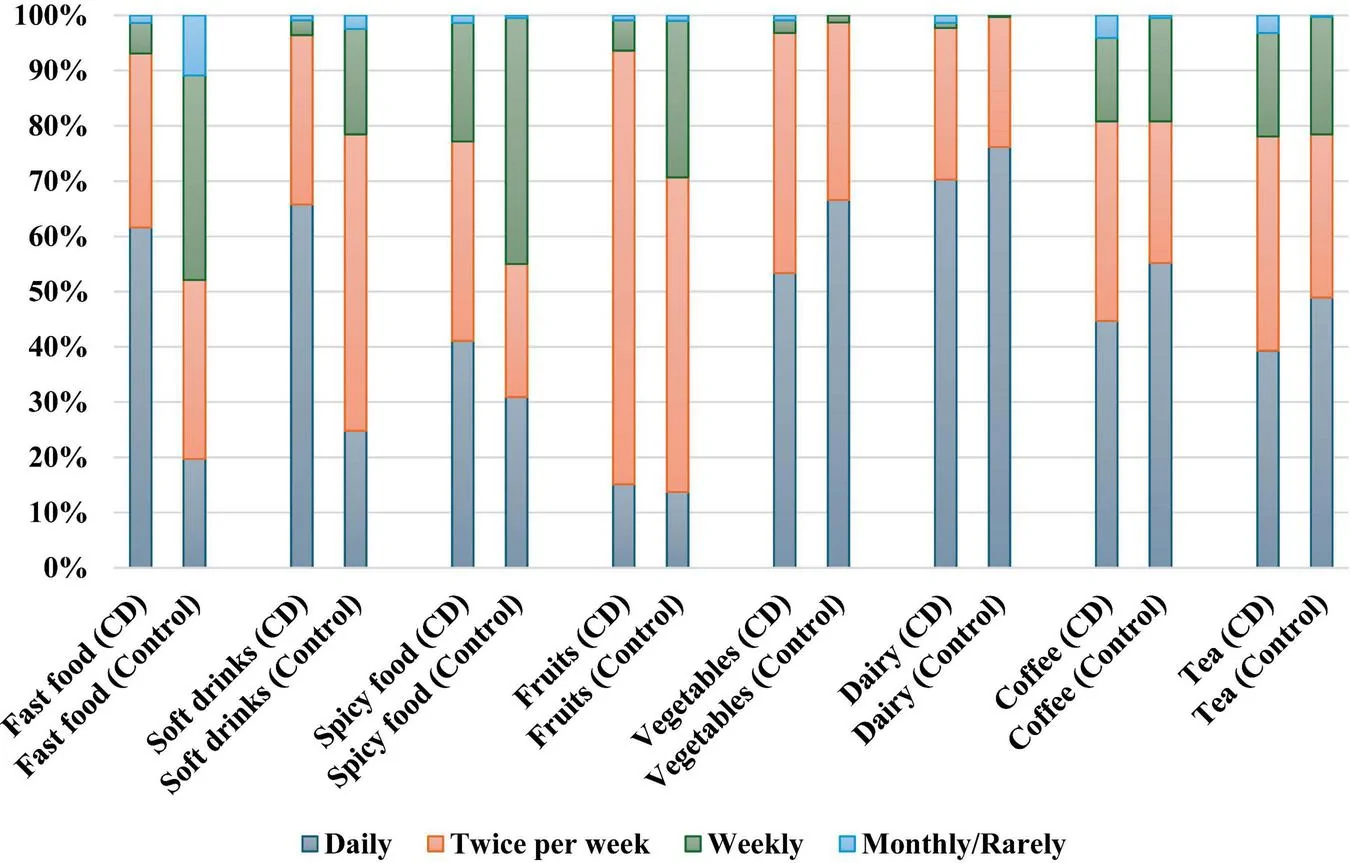
Dietary consumption frequencies among patients with Crohn’s disease and controls.

**FIGURE 2:**
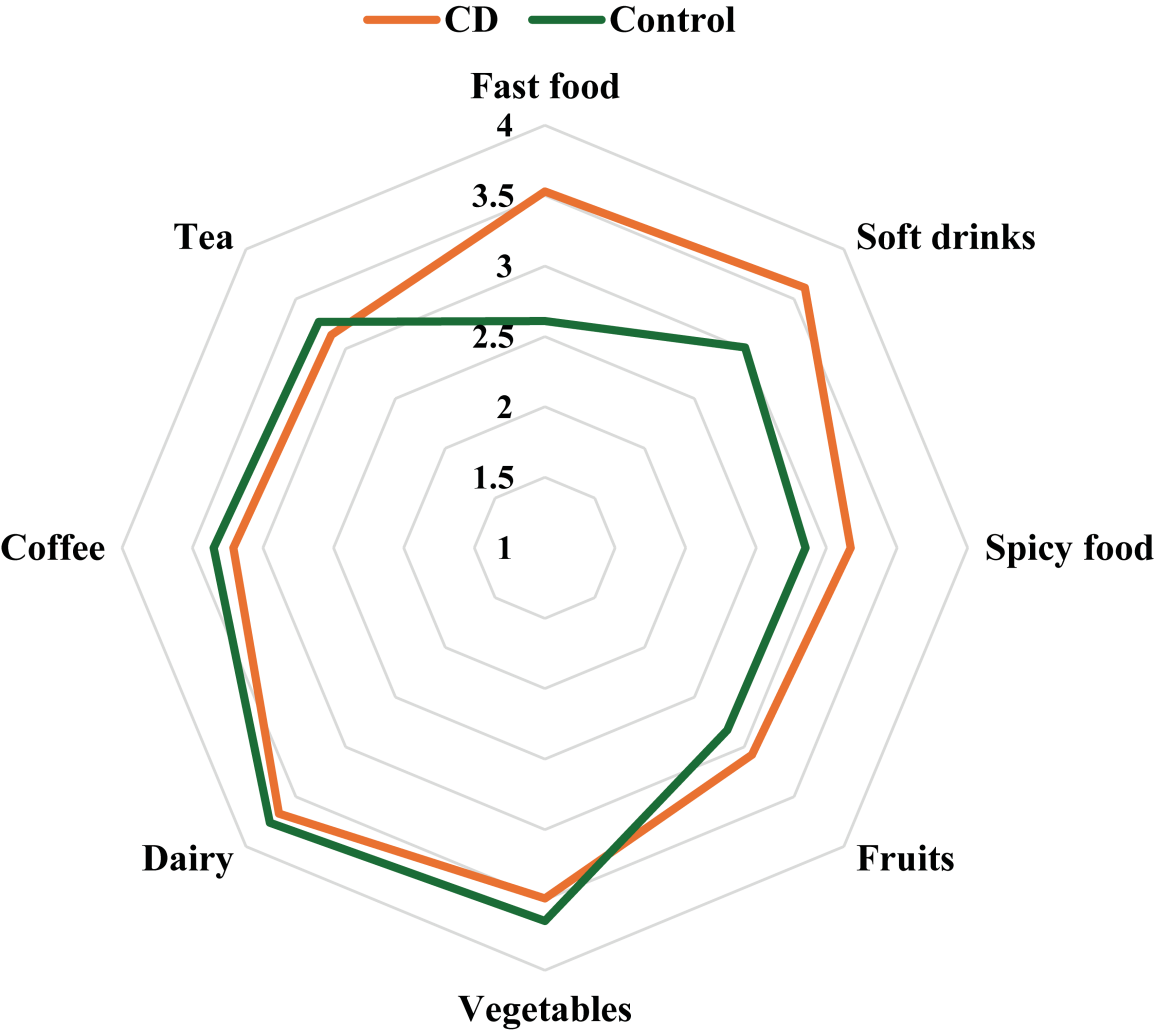
Mean dietary consumption scores among patients with Crohn’s disease and controls.

**TABLE 2:** Dietary consumption scores among patients with Crohn’s disease and controls.

Food items	Crohn’s disease mean ± SD	Control mean ± SD	*P*-value
Fast food	3.53 ± 0.67	2.61 ± 0.92	<0.001
Soft drinks	3.61 ± 0.59	3.01 ± 0.74	<0.001
Spicy food	3.17 ± 0.81	2.85 ± 0.87	<0.001
Fruits	3.08 ± 0.49	2.83 ± 0.66	<0.001
Vegetables	3.49 ± 0.59	3.65 ± 0.50	0.001
Dairy	3.67 ± 0.57	3.76 ± 0.43	0.026
Coffee	3.21 ± 0.85	3.35 ± 0.80	0.038
Tea	3.14 ± 0.83	3.27 ± 0.80	0.052

### Association between individual dietary items and Crohn’s disease

Higher consumption of fast food, carbonated soft drinks, spicy food, and fruits was associated with higher odds of CD across all three models. In the fully adjusted model, each one-unit increase in the fast-food consumption score was associated with more than four-fold higher odds of CD (OR = 4.12, 95% CI: 3.02, 5.63), while the corresponding OR for carbonated soft drinks was 3.89 (95% CI = 2.74, 5.52). Higher spicy-food consumption was associated with 67% higher odds of CD (OR = 1.67, 95% CI: 1.33, 2.09), and higher fruit consumption was associated with more than three-fold higher odds (OR = 3.48, 95% CI: 2.41, 5.02). No significant associations were found for vegetables (OR = 0.85, 95% CI: 0.59, 1.22), dairy products (OR: 0.83, 95% CI: 0.56, 1.24), coffee (OR = 1.01, 95% CI: 0.80, 1.28), or tea (OR = 0.93, 95% CI: 0.73, 1.18) after full adjustment (Table 3).

**TABLE 3:** Association between dietary consumption scores and Crohn’s disease.

Food items	Model I OR (95% CI)	Model II OR (95% CI)	Model III OR (95% CI)
Fast food	3.94 (2.98, 5.22)	3.99 (2.97, 5.37)	4.12 (3.02, 5.63)
Soft drinks	3.68 (2.68, 5.06)	3.95 (2.82, 5.54)	3.89 (2.74, 5.52)
Spicy food	1.50 (1.22, 1.83)	1.61 (1.29, 1.99)	1.67 (1.33, 2.09)
Fruits	3.02 (2.18, 4.20)	3.15 (2.22, 4.46)	3.48 (2.41, 5.02)
Vegetables	0.78 (0.56, 1.08)	0.79 (0.56, 1.11)	0.85 (0.59, 1.22)
Dairy	0.87 (0.62, 1.24)	0.82 (0.56, 1.18)	0.83 (0.56, 1.24)
Coffee	0.87 (0.71, 1.07)	0.94 (0.75, 1.17)	1.01 (0.80, 1.28)
Tea	0.88 (0.70, 1.07)	0.90 (0.72, 1.13)	0.93 (0.73, 1.18)

Model I: adjusted for age and sex; Model II: further adjusted for BMI and smoking; Model III: further adjusted for inflammatory markers.

In sensitivity analyses comparing daily with non-daily consumption, daily consumption of fast food (OR = 6.16, 95% CI: 3.90, 9.72), carbonated soft drinks (OR: 5.38, 95% CI: 3.47, 8.32), spicy food (OR = 1.57, 95% CI: 1.05, 2.33), and fruits (OR: 1.72, 95% CI = 1.00, 2.98) was associated with higher odds of CD. No significant associations were observed for vegetables, dairy products, coffee, or tea (Supplementary Table 1).

### Sex-stratified associations

Higher fast-food and carbonated soft-drink consumption was associated with CD in both men and women. For fast food, the adjusted OR was 3.79 (95% CI: 2.65, 5.43) in men and 5.45 (95% CI: 2.81, 10.57) in women, while the corresponding ORs for carbonated soft drinks were 4.31 (95% CI: 2.80, 6.61) and 3.06 (95% CI: 1.60, 5.82), respectively. Higher fruit consumption was also associated with CD in both men (OR = 3.76, 95% CI: 2.42, 5.84) and women (OR = 3.16, 95% CI: 1.52, 6.57). Higher spicy-food consumption was associated with CD among men (OR = 1.77, 95% CI: 1.35, 2.32), while the estimate among women was less precise (OR = 1.49, 95% CI: 0.97, 2.29). However, there was no evidence of effect modification by sex (*p*-value for interaction > 0.10). No significant associations were found for vegetables, dairy products, coffee, or tea in either sex (Table 4). In sensitivity analyses comparing daily with non-daily consumption, the sex-stratified sensitivity analyses showed broadly similar patterns (Supplementary Table 1).

**TABLE 4:** Sex-stratified associations between dietary consumption scores and Crohn’s disease.

Food items	Men OR (95% CI)	Women OR (95% CI)
Fast food	3.79 (2.65, 5.43)	5.45 (2.81, 10.57)
Soft drinks	4.31 (2.80, 6.61)	3.06 (1.60, 5.82)
Spicy food	1.77 (1.35, 2.32)	1.49 (0.97, 2.29)
Fruits	3.76 (2.42, 5.84)	3.16 (1.52, 6.57)
Vegetables	0.79 (0.56, 1.11)	1.01 (0.66, 1.55)
Dairy	0.81 (0.50, 1.30)	1.04 (0.49, 2.19)
Coffee	0.90 (0.67, 1.19)	1.39 (0.89, 2.17)
Tea	1.05 (0.79, 1.41)	0.74 (0.47, 1.18)

ORs are adjusted for age, BMI, smoking, and inflammatory markers.

### Dietary patterns and Crohn’s disease

The high risk-promoting/low protective dietary pattern was more common among patients with CD than controls (36.5% vs. 17.2%), as was the high risk-promoting/high protective pattern (50.2% vs. 30.4%). In contrast, the low risk-promoting/low protective pattern was less common among patients with CD (4.6% vs. 15.9%), as was the low risk-promoting/high protective pattern (8.7% vs. 36.5%) (Figure 3). Compared with the high risk-promoting/low protective pattern, the low risk-promoting/low protective pattern was associated with substantially lower odds of CD after full adjustment (OR = 0.16, 95% CI: 0.07, 0.35), and a similar association was observed for the low risk-promoting/high protective pattern (OR = 0.14, 95% CI: 0.07, 0.27). The high risk-promoting/high protective pattern was not significantly associated with CD compared with the reference pattern (OR = 1.15, 95% CI = 0.69, 1.92) (Table 5).

**FIGURE 3:**
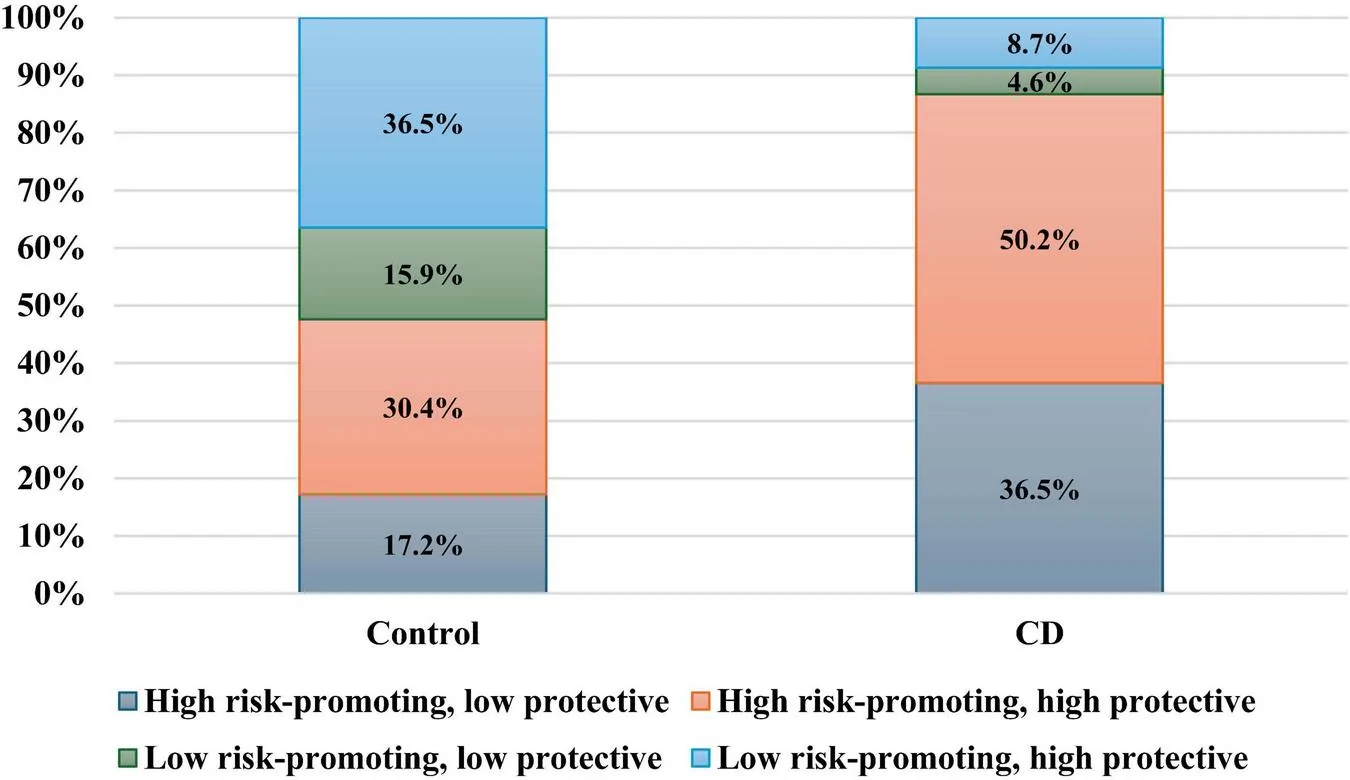
Distribution of combined dietary patterns among patients with Crohn’s disease and controls.

**TABLE 5:** Association between combined dietary patterns and Crohn’s disease.

Dietary patterns	Model I OR (95% CI)	Model II OR (95% CI)	Model III OR (95% CI)
High risk-promoting/low protective	Ref	Ref	Ref
High risk-promoting/high protective	0.89 (0.57, 1.39)	1.02 (0.63, 1.65)	1.15 (0.69, 1.92)
Low risk-promoting/low protective	0.16 (0.07, 0.34)	0.14 (0.07, 0.32)	0.16 (0.07, 0.35)
Low risk-promoting/high protective	0.14 (0.08, 0.27)	0.14 (0.07, 0.27)	0.14 (0.07, 0.27)

Model I: adjusted for age and sex; Model II: further adjusted for BMI and smoking; Model III: further adjusted for inflammatory markers.

## Discussion

The present study aimed to evaluate associations between individual food consumptions and CD, examine combined dietary patterns, and explore potential sex-specific differences. In line with these aims, fast food, carbonated soft drinks, spicy food, and fruit were positively associated with CD, with generally similar findings in men and women. Most importantly, dietary patterns characterized by low consumption of risk-promoting foods were associated with substantially lower odds of CD, irrespective of the level of protective food consumption.

The associations with fast food and carbonated soft drinks are consistent with evidence linking Western and highly processed diets with CD. A meta-analysis of pre-illness dietary patterns found that a Western dietary pattern was associated with higher CD risk (16), while a large prospective study of 245,112 participants reported a 70% higher risk of CD among those in the highest versus lowest quartile of ultra-processed food consumption (18). A meta-analysis of beverage studies also reported a positive association between soft-drink consumption and CD (19). Fast foods and sugar-sweetened beverages may represent several correlated exposures, including refined carbohydrates, added sugars, fats, and food additives. These dietary characteristics may influence intestinal inflammation through changes in immune responses, metabolic pathways, gut microbial composition, and intestinal barrier function (31, 32). The association with spicy food was weaker than those observed for fast food and soft drinks, and the epidemiological evidence for spicy food and incident CD remains limited. Experimental studies indicate that capsaicin can affect TRPV1 signaling, intestinal permeability, and gut microbial composition, although these effects appear to depend on dose and experimental conditions (33, 34). The finding should therefore be interpreted cautiously and does not establish a direct harmful effect of spicy food.

Participants with low risk-promoting consumption had substantially lower odds of CD whether their protective-food consumption was low or high, whereas high protective-food consumption was not associated with lower odds when risk-promoting consumption remained high. This differs from prospective studies in which greater adherence to Mediterranean or other healthy dietary patterns was associated with lower CD risk (22, 23). A recent meta-analysis similarly reported approximately 41% and 30% lower CD risk with Mediterranean and healthy dietary patterns, respectively (35). However, these established dietary patterns incorporate a wider range of foods and dietary characteristics than the protective-food score used in the present study. Our findings therefore suggest that, within the limited set of foods assessed here, lower consumption of risk-promoting foods may have been more strongly associated with CD than higher consumption of the foods classified as protective.

The positive association with fruit was the main finding that differed from the broader literature. Earlier prospective evidence generally suggested an inverse association between fruit consumption and CD (20), whereas the recent meta-analysis found no overall association for combined fruit and vegetable intake, despite a clear inverse association with dietary fiber (35). Most patients with CD were concentrated in the twice-weekly fruit category rather than the daily category, suggesting that the relationship may not follow a simple linear gradient. The positive association with fruit should be considered exploratory and requires confirmation in prospective studies where diet is measured before symptom onset.

The absence of significant associations with vegetables, dairy products, coffee, and tea is also consistent with the variable findings reported for individual foods. Earlier meta-analyses suggested inverse associations of vegetables and dairy products with CD (20, 25), but the more recent synthesis restricted to prospective studies found no significant overall association for vegetables or dairy products (35). Similarly, the available beverage meta-analysis found no association for coffee and an inverse association for tea (19). Taken together, the evidence for individual foods appears less consistent than that for broader dietary patterns or food processing. This may partly reflect differences in dietary assessment, portion size, food preparation, background diet, and the correlations between foods consumed within the same dietary pattern.

The sex-stratified analyses did not suggest major differences between men and women. Fast food, carbonated soft drinks, and fruit were associated with CD in both sexes. Nevertheless, sex-related differences in IBD susceptibility and environmental exposures have been described (29, 30), but prospective evidence for sex-specific dietary associations remains limited. In the Swedish Mediterranean diet cohorts, for example, the association with CD was not modified by sex (22). However, in our recent study from the same dataset, spicy food was positively associated with ulcerative colitis in men, while vegetable consumption in women and dairy consumption in men were inversely associated with ulcerative colitis (36).

Several features strengthen this study. First, CD diagnoses were supported by colonoscopy and histopathology, reducing the possibility of outcome misclassification. Second, dietary information was collected before participants were informed of a confirmed diagnosis, and the use of four consumption categories retained more information than a simple high-versus-low classification. Third, the combined dietary analysis also extends the earlier food-specific analyses by considering selected foods jointly.

The findings should nevertheless be interpreted in light of several limitations. First, the case–control design and retrospective dietary assessment limit inference about temporality. Although participants were asked to report their usual dietary consumption before the onset of gastrointestinal symptoms, dietary information was collected after symptoms had developed; therefore, recall bias and reverse causation cannot be excluded. Second, the use of patients with gastrointestinal disorders as controls may have introduced selection bias, as their symptoms may also have influenced dietary behavior. Third, dietary assessment was limited to consumption frequency and did not include portion size, total energy intake, nutrient composition, or the complete diet. In addition, the questionnaire was not formally validated and its reliability was not assessed, which may have introduced measurement error. Fourth, treating the four frequency categories as an ordinal score assumes a similar change in log odds between consecutive categories, which may not be appropriate for every food. Sensitivity analyses comparing daily with non-daily consumption were therefore performed, although this approach may reduce information on intermediate consumption frequencies. Fifth, the dietary patterns were constructed from the limited dietary variables available in this dataset, and the classification of foods as risk-promoting or protective was based partly on evidence from the same source population. These patterns should therefore be considered operational rather than established dietary indices and require validation in independent populations. Sixth, residual confounding remains possible because information on several potentially relevant dietary, socioeconomic, and lifestyle factors was unavailable. Inflammatory markers were also measured around the time of diagnosis and may themselves have been influenced by active or preclinical CD. Seventh, multiple dietary exposures and sex-stratified analyses were examined without formal correction for multiple comparisons, so some findings, particularly subgroup findings, may have occurred by chance. Eighth, recruitment from a single private clinic in Riyadh and the use of data collected between 2009 and 2017 may limit generalizability to the broader Saudi and Arab populations and to current dietary patterns.

## Conclusion

Frequent consumption of fast food, carbonated soft drinks, and spicy food was associated with higher odds of CD, while dietary patterns characterized by low consumption of risk-promoting foods were associated with markedly lower odds, regardless of protective-food consumption. Given the retrospective assessment of diet, these findings should not be interpreted as evidence of a causal relationship. Prospective studies are needed to confirm these associations and examine potential biological explanations.

## Data Availability

We did not make our data publicly available due to ethical or administrative reasons. Requests to access the datasets should be directed to HS, haytham.sheerah@live.com.
