# Supplementary material for: Food consumption and combined dietary patterns in relation to Crohn’s disease: a case–control study in Saudi Arabia
Source: Front Nutr. 2026 Oct 23;13:1989162. doi: 10.3389/fnut.2026.1989162 (PMC13739521; doi:10.3389/fnut.2026.1989162)
Supplement: Supplementary file 1 [file Table_1.docx]

**Supplementary Table 1: Sensitivity analysis of daily versus non-daily dietary consumption in relation to Crohn’s disease**

| **Food item** | **Overall**  **OR (95% CI)** | **Men**  **OR (95% CI)** | **Women**  **OR (95% CI)** |
| --- | --- | --- | --- |
| Fast food | 6.16 (3.90, 9.72) | 7.20 (4.14, 12.51) | 4.15 (1.74, 9.89) |
| Carbonated soft drinks | 5.38 (3.47, 8.32) | 6.57 (3.84, 11.24) | 3.33 (1.50, 7.41) |
| Spicy food | 1.57 (1.05, 2.33) | 1.64 (1.01, 2.67) | 1.42 (0.69, 2.93) |
| Fruits | 1.72 (1.00, 2.98) | 1.78 (0.91, 3.47) | 1.58 (0.57, 4.36) |
| Vegetables | 0.89 (0.59, 1.34) | 1.14 (0.68, 1.82) | 0.58 (0.26, 1.29) |
| Dairy products | 0.94 (0.60, 1.45) | 0.92 (0.54, 1.57) | 1.14 (0.50, 2.60) |
| Coffee | 0.86 (0.58, 1.26) | 0.65 (0.41, 1.04) | 1.61 (0.77, 3.34) |
| Tea | 0.70 (0.47, 1.05) | 0.79 (0.50, 1.25) | 0.45 (0.18, 1.10) |

Non-daily consumption included twice-weekly, weekly, and monthly/less consumption. The overall model was adjusted for age, sex, BMI, smoking, and inflammatory markers. Sex-stratified models were adjusted for age, BMI, smoking, and inflammatory markers
